# Morphology-aware distillation for lightweight retinal vessel segmentation across fundus photography and OCT angiography

**DOI:** 10.3389/fcell.2026.1825518

**Published:** 2026-05-28

**Authors:** Han Shu, Qi Yuan, Yizhi Pan, Weikun Kong, Yangyi Feng, Cheng Peng, Kai Li, Le-Minh Nguyen, Teeradaj Racharak, Junyi Xin, Guanqun Sun

**Affiliations:** 1 School of Information Engineering, Zhejiang Provincial People’s Hospital, Affiliated People’s Hospital, Hangzhou Medical College, Hangzhou, China; 2 School of Information Science, Japan Advanced Institute of Science and Technology, Nomi, Japan; 3 Department of Electronic Engineering, Tsinghua University, Beijing, China; 4 Advanced Institute of So-Go-Chi (Convergence Knowledge) Informatics, Tohoku University, Sendai, Miyagi, Japan

**Keywords:** deep learning, lightweight segmentation, morphology-aware distillation, ophthalmology, retinal vessel segmentation

## Abstract

**Introduction:**

Retinal vessel segmentation is critical for diagnosing ophthalmic and systemic diseases, yet deploying high-performance models in resource-constrained clinical settings remains a challenge. While Knowledge Distillation (KD) offers a solution for model compression, conventional KD methods often treat the U-Net as a generic feature extractor, neglecting the unique topological nature of vascular networks. This oversight frequently leads to “fractured” segmentation maps in student models, where fine capillaries and continuous vessel branches are lost.

**Methods:**

In this paper, we advocate a task-specific, morphology-aware distillation strategy for lightweight retinal vessel segmentation. We introduce Morphology-Aware Reconstruction Distillation (MRD), a novel framework designed to transfer the teacher’s capability to reconstruct coherent vascular graphs rather than merely mimicking pixel statistics. Central to MRD is the Hierarchical Structure Fusion (HSF) module, a purpose-built unit that adaptively integrates multi-scale features using a tailored residual gating mechanism. By focusing on the decoder’s role as a topological reconstruction engine, HSF guides the student to learn how to synthesize continuous vessel structures from compressed representations. We validate our approach across two distinct modalities: Fundus Photography (FIVES and DRIVE datasets) and OCT Angiography (ROSE dataset).

**Results:**

Extensive experiments demonstrate that our method significantly outperforms existing KD techniques. Notably, our distilled student model achieves diagnostic-level segmentation fidelity that surpasses massive teacher architectures like TransUNet, all while operating with a radically reduced parameter footprint.

**Discussion:**

By robustly preserving delicate vascular morphology across diverse datasets, these results highlight the strong translational potential of our approach, providing a structurally reliable and highly efficient solution tailored for point-of-care clinical deployment. The code will be released.

## 1 Introduction

The retinal microvasculature is a critical, non-invasive biomarker for the early diagnosis and personalized treatment planning of systemic and ocular diseases, precisely aligning with the imperative to integrate AI into foundational ophthalmic research (Leitgeb, 2019; Alnawaiseh et al., 2018). Translating AI into actual clinical workflows relies heavily on two complementary imaging modalities: Color Fundus (CF) photography for widespread 2D screening (Van Grinsven et al., 2016), and Optical Coherence Tomography Angiography (OCTA) for depth-resolved capillary visualization (Yoon et al., 2019). Therefore, developing automated, highly accurate segmentation algorithms capable of operating across both modalities is the cornerstone of modern computational ophthalmology.

**FIGURE 1:**
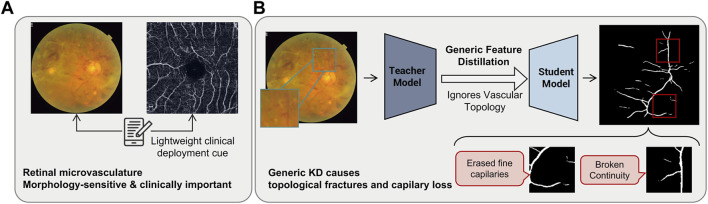
Conceptual illustration of lightweight clinical deployment in ophthalmology. The translation of automated retinal vessel segmentation into real-world clinical workflows demands deploying high-performance algorithms directly onto resource-constrained edge devices, such as portable fundus cameras and point-of-care OCTA systems. This necessitates highly compact yet morphologically accurate models to ensure reliable on-site diagnostics. **(A)** Ophthalmic Motivation & Dual-Modality Context. **(B)** Why Generic Distillation Fails.

While deep learning architectures, ranging from standard U-Nets (Ronneberger et al., 2015; Diakogiannis et al., 2020; Lian et al., 2018) to advanced Transformers (Chen J. et al., 2021; Cao et al., 2022), have achieved remarkable segmentation accuracy, their massive computational requirements create a severe “deployment gap”. In real-world ophthalmology, there is an urgent demand to deploy these diagnostic tools directly into resource-constrained clinical settings, such as portable fundus cameras and point-of-care OCTA devices (as in Figure 1a). To bridge this gap, Knowledge Distillation (KD) (Hinton et al., 2015) offers a theoretical pathway by compressing heavy “teacher” networks into lightweight “student” models, aiming to achieve edge deployment without sacrificing clinical accuracy (Chen P. et al., 2021; Liu et al., 2023a).

However, standard KD strategies often falter when applied to the unique structural demands of ophthalmic imaging. Specifically, applying generic distillation to retinal vessel segmentation across diverse modalities faces two primary challenges: First, topological blindness. Conventional distillation typically treats semantic segmentation as a set of independent pixel-wise classifications (Liu et al., 2019), neglecting a fundamental biological prior: retinal vessels form continuous, topological graphs (Mosinska et al., 2018). This pixel-centric oversight leads to severe morphological degradation, causing “topological fractures” in the student model (Shit et al., 2021) which manifests as broken continuous branches in CF and erased fine capillaries in OCTA, as explicitly illustrated in Figure 1b. Second, inefficient hierarchical reconstruction. Lightweight student models inherently struggle with a limited capacity to fuse high-level semantics with low-level spatial details (Mou et al., 2019). This multi-scale fusion is essential for isolating thin vessels from profound modality-specific noise, such as the severe illumination variance and low contrast in CF imaging (Jin et al., 2022), as well as the inherent projection artifacts in depth-resolved OCTA scans (Ma et al., 2021). Standard KD typically applies a diluted, whole-network feature mimicry, failing to specifically guide the student’s decoder on how to effectively weave these multi-scale hierarchical features together to resist such ophthalmic imaging artifacts.

To overcome these limitations, an effective distillation framework for ophthalmic imaging must move beyond generic model compression. From a biological perspective, the distillation process must strictly preserve the morphological fidelity and topological connectivity of the vascular networks. From an architectural perspective, the lightweight student network requires explicit guidance during the decoding phase to efficiently integrate hierarchical features, ensuring that delicate capillary structures are not lost to modality-specific noise.

Guided by these biological and structural imperatives, we propose a novel framework: Morphology-Aware Reconstruction Distillation (MRD). To specifically address the challenge of topological blindness, MRD shifts the distillation focus away from independent feature imitation and exclusively targets the network’s decoder. By teaching the student the structural “grammar” of vessel connectivity, MRD ensures a topologically sound segmentation that respects the biological priors of the retina. Furthermore, to resolve the bottleneck of inefficient hierarchical reconstruction, we introduce the Hierarchical Structure Fusion (HSF) module as the core engine of our framework. HSF employs an adaptive residual gating mechanism to selectively fuse multi-scale features. This targeted design explicitly guides the student’s upsampling process, effectively filtering out background noise while rigorously preserving high-frequency capillary details.

In summary, the main contributions of this paper are as follows:

1. We introduce Morphology-Aware Reconstruction Distillation (MRD), a task-specific decoder distillation that tackles topological blindness by targeting the decoder’s reconstruction capabilities, effectively preventing vessel fragmentation.
2. We propose the Hierarchical Structure Fusion (HSF) module, a specialized architectural unit designed to address the inefficient hierarchical reconstruction in lightweight networks, significantly enhancing multi-scale feature reconstruction.
3. We validate our proposed framework on two distinct modalities: the Fives dataset, Drives dataset (Fundus Photography) and the Rose dataset (OCTA). Experimental results demonstrate that our method significantly outperforms existing distillation techniques, achieving favorable trade-offs between computational efficiency and morphological accuracy across both imaging types.

## 2 Related work

### 2.1 Retinal vessel segmentation in ophthalmic imaging

The precise segmentation of retinal microvasculature is a fundamental prerequisite for diagnosing ophthalmic diseases. While generic ophthalmic image segmentation architectures—ranging from the foundational U-Net (Ronneberger et al., 2015) to recent Transformer-based models (Chen J. et al., 2021; Cao et al., 2022) have achieved impressive accuracy, retinal vessel segmentation presents unique topological challenges.

To address this, specialized networks have been proposed. For Color Fundus (CF) images, models like SA-UNet (Guo et al., 2021) and IterNet (Li et al., 2020) utilize spatial attention and iterative refinement to capture elongated vascular structures. For Optical Coherence Tomography Angiography (OCTA), methods such as OCTA-Net (Ma et al., 2021) are explicitly designed to delineate complex, depth-resolved capillary networks. However, the pursuit of higher accuracy across these heterogeneous modalities has inadvertently led to the proliferation of massive, computationally expensive models. Current research largely ignores the critical need for parameter efficiency, creating a significant barrier for real-time deployment in resource-constrained clinical settings.

### 2.2 Efficient and lightweight ophthalmic segmentation

To deploy automated ophthalmic analysis on edge devices, such as portable fundus cameras and point-of-care OCTA systems, researchers have actively explored various lightweight network architectures explicitly designed for retinal imaging. For instance, compact architectures like Wave-Net (Liu et al., 2023b) have been proposed to minimize computational overhead while preserving thin vessels in Color Fundus (CF) images. Similarly, for Optical Coherence Tomography Angiography (OCTA), lightweight full-resolution architectures like FRNet V2 (Gao et al., 2025) have been developed to accelerate inference speeds and reduce memory footprints without sacrificing intricate capillary details. While designing lightweight architectures from scratch is an effective paradigm, Knowledge Distillation (KD) (Hinton et al., 2015) has emerged as an equally vital, orthogonal strategy to compress massive, high-performance teacher models into compact student networks. Early KD approaches primarily focused on matching final output probabilities or implementing generic regularizations (Cho and Hariharan, 2019). Subsequent advancements shifted toward intermediate feature-based distillation (Romero et al., 2015), attention map transfer (Yang et al., 2023), and relational knowledge alignment (Park et al., 2019).

However, literature addressing KD specifically tailored for the retinal microvasculature remains limited. A critical technical limitation of current generic distillation paradigms is their “topology-blind” nature. Existing methods typically formulate intermediate distillation as a localized, pixel-wise feature alignment problem, neglecting the continuous, graph-like biological priors of vascular networks. Consequently, lightweight student models distilled via generic feature mimicry often suffer from severe morphological degradation. Without explicit structural guidance during the reconstruction process, these models are prone to producing fragmented major vascular branches in CF photography and failing to delineate delicate capillary beds in OCTA scans. In contrast to existing generic KD methods that perform whole-network spatial alignment, our work diverges by focusing specifically on the decoder’s reconstruction phase. By explicitly guiding the hierarchical fusion of features, we address the morphological shortcomings present in current lightweight ophthalmic segmentation literature.

## 3 Methodology

### 3.1 Theoretical intuition and problem definition

**FIGURE 2:**
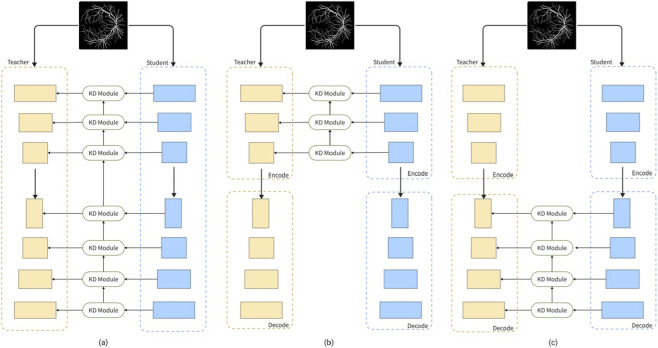
The architecture of three kinds distillation. **(a)** Normal Distillation: Distillation of all features. **(b)** Encoder Distillation: Distillation of encoder features. **(c)** Decoder Distillation: Distillation of decoder features. The Encode layers represent the outputs of each encoding stage, capturing hierarchical features through downsampling. The Decode layers correspond to the outputs of each decoding stage, enhanced by skip connections that integrate corresponding encoder features.

While conventional Knowledge Distillation (KD) paradigms have demonstrated success in model compression, they predominantly anchor the distillation loss at all stages (Figure 2a) or specifically at the deep encoder stages (Figure 2b). However, in the context of retinal vessel segmentation, preserving the morphological and topological continuity of microvasculature is strictly paramount. The primary mathematical function of an encoder is spatial compression and semantic abstraction. This process inherently discards high-frequency spatial correlations, making encoder-centric distillation fundamentally ill-suited for topology preservation. Conversely, the decoder is dedicated to progressive spatial reconstruction. The topological integrity of a vessel is defined by the spatial gradients and local pixel affinities within the high-resolution feature space. Therefore, as illustrated in Figure 2c, we hypothesize that shifting the distillation focus exclusively to the decoder uniquely preserves these structural properties.

Formally, the goal of our study is to achieve high-fidelity retinal vessel segmentation in resource-constrained clinical settings. We define this task as a morphology-preserving reconstruction problem. Let $x\in R^{W\times H\times C}$ denote an input ophthalmic image, $y_{s}\in {\left\{0,1\right\}}^{W\times H}$ denote the student network’s generated vascular map, and $Y\in {\left\{0,1\right\}}^{W\times H}$ denote the corresponding Ground Truth. Our objective is to train a lightweight student network S that generates a prediction $y_{s}$ while preserving the delicate vascular continuity typically only captured by a high-performance teacher network T (e.g., TransUNet).

To ensure the architectural homology required for preserving vascular morphology, both T and S follow a U-shaped topology. Each model can be decomposed into an encoder-decoder sequence consisting of m stages. We denote the latent feature representation at stage i as $F_{i}$ . The forward propagation process from the input x to the final vessel segmentation mask $y_{s}$ can be formulated as follows in Equation 1:

$$\begin{array}{cc} & F_{1}\left(x\right)=g_{1}\left(x\right),\\ & F_{2}\left(x\right)=g_{2}\left(F_{1}\left(x\right)\right)=g_{2}\left(g_{1}\left(x\right)\right),\\ & \ldots \ldots ,\\ & F_{m}\left(x\right)=g_{m}\left(F_{m-1}\left(x\right)\right),\\ & y_{s}=c\left(F_{m}\left(x\right)\right). \end{array}$$ (1)

where $g_{i}\left(\cdot \right)$ represents the i -th transformation stage (comprising convolution, pooling, and activation) and c(⋅) is the final pixel-wise classifier. Unlike generic segmentation tasks, the vascular reconstruction in $y_{s}$ is highly sensitive to the spatial information embedded in the intermediate decoder layers.

To bridge the capacity gap between the teacher features $F_{i}^{t}$ and student features $F_{i}^{s}$ at the decoder stages, while preventing vessel fragmentation, we introduce a hierarchical transformation f(⋅) representing our Hierarchical Structure Fusion (HSF) module. This process aligns and recalibrates the student’s multi-scale features as follows in Equation 2:

$$\begin{array}{cc} & {\tilde{F}}_{m}=f_{m}\left(F_{m}\right),\\ & {\tilde{F}}_{m-1}=f_{m-1}\left(F_{m-1},{\tilde{F}}_{m}\right),\\ & \ldots \ldots ,\\ & {\tilde{F}}_{1}=f_{1}\left(F_{1},{\tilde{F}}_{2}\right), \end{array}$$ (2)

where ${\tilde{F}}_{i}$ denotes the enhanced feature representation at stage i . Finally, our Morphology-Aware Reconstruction Distillation (MRD) objective minimizes a joint loss function that balances traditional semantic overlap with task-specific structural fidelity as follows in Equation 3:

$$L_{total}=\alpha \times \overset{m}{\underset{i=0}{\sum }}L_{fm}\left({\tilde{F}}_{i}^{s},F_{i}^{t}\right)+\left(1-\alpha \right)\times L_{seg}\left(y_{s},Y\right)$$ (3)

**FIGURE 3:**
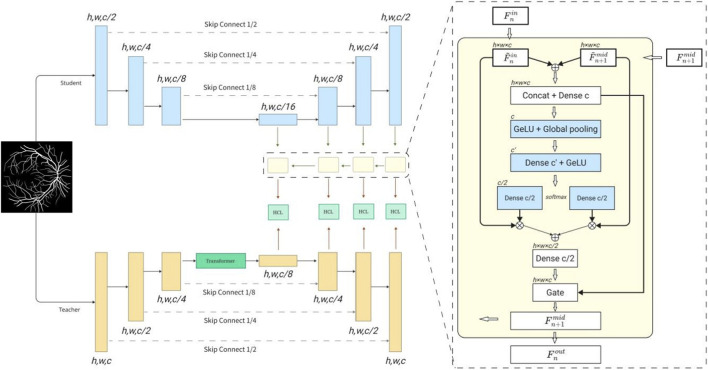
Overall framework of the proposed MRD. It is composed of two parts: the decoder distillation, and distillation branches in features. The Decoder Distillation framework, shown in Figure 2c, distills only the decoder feature layers of the U-Net model, leveraging skip connections to reconstruction spatial and structural knowledge from teacher to student. In each branch, the transformation from student features to teacher features is facilitated by a HSF module and a HCL loss module. HSF fuses the $F_{i}^{s}$ and the next branch ${\tilde{F}}_{i+1}^{s}$ into ${\tilde{F}}_{i}^{s}$ , which learn from the teacher feature $F_{i}^{t}$ and output to the previous feature $F_{i-1}^{s}$ .

Here, $L_{seg}$ denotes the standard segmentation loss (e.g., Dice loss), and α∈(0,1) is a balancing hyperparameter, empirically set to 0.5, to weigh the segmentation loss against the distillation loss. $L_{fm}$ represents the feature map distillation loss between the i -th decoder stage of the teacher and the student. By strictly applying this loss calculation method at the decoder, we strike an optimal balance between the ground truth labels and the morphological knowledge transferred from the teacher. The detailed framework diagram is illustrated in Figure 3.

### 3.2 Morphology-aware reconstruction distillation (MRD)

Instead of indiscriminately applying Knowledge Distillation (KD) across all stages of the neural network, we argue that a biologically meaningful paradigm must strategically target the decoding phase. In the study of retinal microvasculature, preserving the precise architecture of capillary networks is essential for understanding pathological vascularization and cellular-level ischemic changes (Diakogiannis et al., 2020; Chen J. et al., 2021; Cao et al., 2022; Wang et al., 2024). In this context, the U-shaped architecture is highly prized for its unique capacity to reconstruct these delicate spatial details. While the encoder focuses on broad semantic abstraction (e.g., distinguishing general vascular regions from the retinal background), the decoder functions as the critical engine for fine-grained morphological reconstruction. Through up-sampling and skip-connections, it integrates spatial cues to rebuild continuous, biologically coherent vascular trees. Therefore, isolating distillation specifically to the decoder forces the student model to learn this intricate reconstruction process, directly preventing the artificial fragmentation of capillaries that plagues standard model compression methods.

Our goal is to improve lightweight retinal vessel segmentation by distilling the reconstruction process that preserves vascular continuity. Conventional KD methods (Chen P. et al., 2021; Liu et al., 2023a) typically enforce rigid, layer-to-layer feature mimicry, treating the vascular network as mere pixel patterns rather than continuous biological structures. Drawing inspiration from efficient distillation strategies (Qi et al., 2022; Bai et al., 2023), we propose a targeted framework that respects the structural continuity of the vasculature. Unlike previous approaches, our MRD framework concentrates constraints exclusively on the decoder’s hierarchical feature maps. By employing specialized HSF modules at key reconstruction intersections, we guide the lightweight student model to synthesize vascular networks progressively—from coarse anatomical landmarks down to delicate capillary beds. This decoder-centric approach not only drastically reduces computational overhead for potential point-of-care deployment but, crucially, helps preserve morphology relevant to retinal vascular analysis of the extracted vascular biomarkers across both Fundus and OCTA imaging modalities.

1. *Normal Distillation.* Like all previously proposed relation-based distillation methods (Cho and Hariharan, 2019; Qi et al., 2022; Yang et al., 2022), all the feature layers of students is reconstructed as the teacher’s, which is shown in Figure 2a. As the normal KD framework, the distillation loss penalty function for the entire U-Net network $Loss_{fm}=Loss_{fm,Encode}+Loss_{fm,Decode}$ in Equation 4, where m1 is the number of feature maps in Encoder and m2 is he number of feature maps in as follows in Equation 4:

$$Loss_{fm=\overset{m1+m2}{\underset{i=0}{\sum }}L_{fm}\left({\tilde{F}}_{i}^{s},F_{i}^{t}\right)=\overset{m1+m2}{\underset{i=0}{\sum }}L_{fm}\left(HSF\left(F_{i}^{s},{\tilde{F}}_{i+1}^{s}\right),F_{i}^{t}\right)}$$ (4)

2. *Encoder Distillation.* Based on the Down-sampling of the U-shaped model, the customized EncoderKD distills only the features of the encoder in the U-shaped model, as shown in Figure 2b, distillation is performed in the Encoder layer before the Transformer module in Figure 3 as follows in Equation 5:

$$Loss_{fm,Encode}=\overset{m1}{\underset{i=0}{\sum }}L_{fm}\left({\tilde{F}}_{i}^{s},F_{i}^{t}\right)$$ (5)

3. *Decoder Distillation.* Based on the Up-sampling and Skip-Connection features of the U-shaped model, focusing on Decoders with both global vision and cross-stage features, we distill only the features of the decoder in Figure 2c, distillation is performed in the Decoder layer after the Transformer module in Figure 3 as follows in Equation 6:

$$Loss_{fm,Decode}=\overset{m1+m2}{\underset{i=m1+1}{\sum }}L_{fm}\left({\tilde{F}}_{i}^{s},F_{i}^{t}\right),F_{i}=f\left(F_{i-1}+F_{i}^{\mathrm{skip}}\right)=f\left(F_{i-1}+F_{m1+m2-i}^{\mathrm{Encode}}\right)$$ (6)

where the f represents the fusion method of the decoded features and the encoded features from the skip connections, which consists of a concatenation operation followed by two convolutional layers.

In this paper, our structure employs Decoder distillation, as shown in Figure 3, where HSF, introduced in the next section, is designed to enhance our U-shaped structure models. We also designed the efficiency of the KD module in U-shaped structure experiments in Section 4.4.2.1 to demonstrate the efficiency of our proposed framework.

### 3.3 Hierarchical structure fusion (HSF)

The Hierarchical Structure Fusion (HSF) module serves as the architectural engine driving our proposed Morphology-Aware Reconstruction Distillation (MRD) paradigm. Traditional knowledge distillation treats the U-Net decoder as a black box, forcing the student to blindly mimic the teacher’s output representations. This naive feature mimicry inevitably leads to morphological degradation in complex vascular networks. To address this, HSF is meticulously engineered to distill the teacher’s underlying “synthesis methodology” teaching the student *how* to weave together multi-level hierarchical information to reconstruct continuous topologies.

**FIGURE 4:**
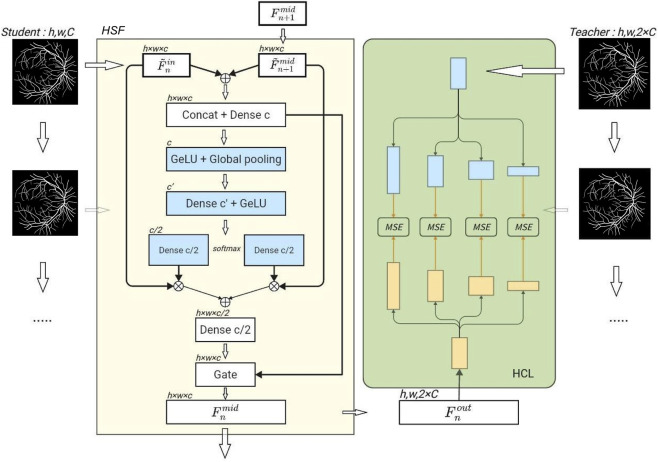
Illustration of the Hierarchical Fusion (HSF) module and the Holistic-level Contrastive Learning (HCL) loss. (Left) The HSF module architecture. It takes student features from the current stage $\left(S_{i}\right)$ and the previous stage $\left(S_{i-1}\right)$ as input. The “Fuse Cross Stage Feature” component generates initial fused features. “Selective Fusion” then computes attention weights to adaptively recalibrate the importance of these features. Finally, the “Residual Gating” mechanism combines the recalibrated features with the original student features $\left(S_{i}\right)$ to produce the distilled output, which is supervised by the teacher’s features $\left(T_{i}\right)$ . This process enables the student to learn the teacher’s feature synthesis strategy. (Right) The HCL loss mechanism. It treats the entire feature maps from the teacher and student as holistic representations (positive pairs) and contrasts them with feature maps from other stages or models (negative pairs, not explicitly shown), encouraging structural similarity at a global level.

As illustrated in Figure 4 (Left), HSF accomplishes this through three synergistic components: *Fuse Cross Stage Feature* (FCSF), *Selective Fusion* (SF), and *Residual Gating* (RG). First, the FCSF and SF modules work in tandem to resolve the inherent conflict between high-frequency spatial details (crucial for delicate capillaries) and high-level semantic context (necessary for background noise suppression). Drawing inspiration from selective attention paradigms (Li et al., 2019; Zhang et al., 2022; Ren et al., 2022), we adapt these mechanisms specifically for the generative decoding process. Instead of static feature concatenation, our Selective Fusion dynamically recalibrates the channel-wise importance of features from different stages. This establishes a shared contextual understanding, guiding the student to identify which hierarchical cues are most salient for preserving vascular connectivity.

Furthermore, aggressive feature fusion during distillation can inadvertently overwrite the student’s intrinsic local representations. To mitigate this, we introduce the RG mechanism. Rather than a simple addition, this mechanism acts as an adaptive knowledge filter, intelligently blending the newly distilled hierarchical features with the student’s original spatial features. This ensures that the distillation process enriches the student’s global structural awareness without sacrificing the fine-grained, modality-specific details already learned. In essence, HSF provides a structured pathway for the student to internalize complex topological reconstruction, ensuring morphology-preserving segmentation across both CF and OCTA modalities.

Fuse Cross Stage Feature: This module obtains preliminary fusion features by fusing student features from both phases, which are then processed by the Selective Fusion module. It mainly consists of a global pooling layer $f_{gp}$ and two pairs of fully connected layers $f_{fc}$ and activation layers $f_{a}$ , as follows in Equations 7,8:

$${\tilde{F}}_{i}^{\mathrm{mid}}=f_{fc}\left({\tilde{F}}_{i}^{in}+F_{i+1}^{\mathrm{mid}}\right)$$ (7)

$$y_{\mathrm{fuse}}=f_{a}\left[f_{fc}\left({\tilde{y}}_{\mathrm{fuse}}\right)\right],{\tilde{y}}_{\mathrm{fuse}}=f_{a}\left({\tilde{F}}_{i}^{\mathrm{mid}}\right),$$ (8)

where the specific dimension setting is presented in Figure 4, where $y_{\mathrm{fuse}}\in R^{1\times 1\times c^{\prime }}$ and $C^{\prime }$ is set to $\frac{C}{4}$ .

Selective Fusion: After this module gets the processed compact features, it is processed by softmax as two channels with the number of channels corresponding to the channel soft attention of $\frac{C}{2}$ . The softmax layer generates corresponding weights for ${\tilde{F}}_{i}^{in}$ and ${\tilde{F}}_{i+1}^{\mathrm{mid}}$ as follows in Equations 9,10:

$$a_{c}=\frac{e^{A\cdot y_{\mathrm{fuse}}}}{e^{A\cdot y_{\mathrm{fuse}}}+e^{B\cdot y_{\mathrm{fuse}}}},b_{c}=\frac{e^{B\cdot y_{\mathrm{fuse}}}}{e^{A\cdot y_{\mathrm{fuse}}}+e^{B\cdot y_{\mathrm{fuse}}}},a_{c}+b_{c}=1,$$ (9)

$$y_{\mathrm{select}}=a_{c}\cdot {\tilde{F}}_{i}^{in}+b_{c}\cdot {\tilde{F}}_{i+1}^{\mathrm{mid}}$$ (10)

where $y_{\mathrm{select}}\in R^{h\times w\times \left(c/2\right)}$ is the fused feature and $A,B\in R^{C/2}$ are two learnable matrices. Then, we use a convolution to get ${\tilde{y}}_{\mathrm{select}}\in R^{h\times w\times c}$ , which is then residual gated. The new selected feature obtained after selecting the two features by Softmax is then processed with the initial feature by the *Residual Gating* mechanism.

Residual Gating: This module gates the fused and unfused features to ensure that the original features are maximally preserved. Finally, the HSF module output is as follows in Equation 11:

$$gate=\delta \left({\tilde{y}}_{\mathrm{select}},{\tilde{F}}_{i}^{\mathrm{mid}}\right),F_{i}^{\mathrm{mid}}=gate*{\tilde{F}}_{i}^{\mathrm{mid}}+\left(1-gate\right)*y_{\mathrm{select}}$$ (11)

Futher, we conduct a 3 × 3 convolution to convert $F_{i}^{\mathrm{mid}}$ to $F_{i}^{\mathrm{out}}$ , which has the same dimension as the teacher’s feature map, where δ is sigmoid function and ∗ denote the Element-wise multiplication. Moreover, $F_{i}^{\mathrm{mid}}$ is the next HSF’s input $F_{i+1}^{\mathrm{mid}}$ , and $F_{i}^{\mathrm{out}}$ will be used in HCL, as seen in Figure 4.

Feature Map Loss Function: During the feature-based and relation-based training processes, we calculate the HCL (Hierarchical Context Loss) (Chen P. et al., 2021) by comparing the outputs with those of the teacher transformer block at each stage in Figure 3 and in Figure 4. The feature map obtained at each stage, with a shape of [b, c, h, w], is segmented into 4-level context information through adaptive average pooling. The 4-level stages are divided into [b,c,h,w], [b,c,4,4], [b,c,2,2] and [b,c,1,1]. At each stage, L2 distances are used to distill knowledge between context levels, and then each stage is separately distilled using the L2 distance. The ultimate HCL loss between the feature maps at the n -th stage is computed as follows in Equation 12:

$$L_{fm}=\frac{1}{1+\underset{l}{\sum }\frac{1}{2^{l}}}\overset{3}{\underset{l=0}{\sum }}\frac{1}{2^{l}}MSE\left(F_{i,l}^{\mathrm{out}},F_{i,l}^{t}\right)$$ (12)

In Figure 4, we detail how the student feature maps are reconstructed to match the spatial dimensions of the teacher feature maps, and how the distillation loss is calculated. Specifically, we enhance the accuracy and efficiency of the U-shaped segmentation model by integrating the proposed HSF module and HCL loss into our lightweight retinal vessel segmentation framework. Compared to existing knowledge distillation methods such as Attention-Based Fusion (ABF) and Cross Selective Fusion (CSF), our HSF module offers several distinct advantages. While both ABF and CSF aim to fuse and select features from different stages, HSF introduces a more comprehensive approach through its three-stage mechanism. First, the cross-stage feature fusion component allows for better integration of multi-scale information, addressing limitations in standard feature aggregation. Second, the selective fusion stage, with its softmax-based weighting, provides a dynamic selection mechanism tailored for the decoder’s synthesis task, enabling a better balance between semantic and spatial information compared to generic fusion methods. Finally, the residual gating mechanism ensures that original feature information is preserved, an aspect often overlooked in existing approaches. This comprehensive design enables HSF to capture more complex spatial dependencies in the feature space, ultimately driving more effective knowledge reconstruction. The superiority of HSF compared to these baselines is quantitatively demonstrated through our experimental results.

## 4 Experiments and analysis

### 4.1 Datasets

Fundus image dataset for vessel segmentation dataset(Fives, (Jin et al., 2022)): the dataset represents the largest public collection of high-resolution retinal images designed for vessel segmentation tasks to date. It consists of 800 color fundus photographs with a resolution of 2048 × 2048 pixels. The dataset covers a diverse range of conditions to ensure robustness, including Normal eyes, Diabetic Retinopathy (DR), Age-related Macular Degeneration (AMD), and Glaucoma. The data distribution is balanced, with 200 images allocated to each of the four categories.

Retinal OCT-Angiography vessel segmentation dataset(Rose, (Ma et al., 2021)): The ROSE dataset is utilized as the OCTA data source in this study. It comprises 39 superficial OCTA fundus images with a resolution of 304 × 304 pixels. Following the official partition scheme, the dataset is divided into a training set of 30 images and a testing set of 9 images.

Color fundus vessel segmentation dataset [Digital Retinal Images for Vessel Extraction, DRIVE (Staal et al., 2004)]: The DRIVE dataset is employed to evaluate the model’s cross-center generalizability on color fundus photography. It consists of 40 color fundus images with a resolution of 768 × 584 pixels. Following the standard official partition, the dataset is evenly divided into a training set of 20 images and a testing set of 20 images.

### 4.2 Evalution metrics

To comprehensively evaluate our morphology-preserving framework, we employ a modality-tailored metric strategy. For our primary evaluation on Color Fundus images (FIVES dataset), we utilize the Dice Similarity Coefficient (DSC), mean Intersection over Union (mIoU), Hausdorff Distance (HD), and Average Surface Distance (ASD) to rigorously assess both global region agreement and boundary fidelity. Conversely, for evaluations on the DRIVE and ROSE datasets, we specifically focus on robust region-based metrics: DSC and mIoU. This distinction is primarily driven by the unique characteristics of these datasets. For OCTA (ROSE), the dense capillary plexuses and inherent projection artifacts render boundary-distance metrics (HD/ASD) mathematically unstable and hypersensitive to pixel-level noise. For the widely established DRIVE benchmark, reporting DSC and mIoU aligns with the standardized evaluation protocols predominantly used in existing literature, ensuring a direct and objective comparison against a broader range of state-of-the-art methods.

DSC (Dice Coefficient) is a metric for evaluating model performance in image segmentation tasks, especially beneficial for addressing class imbalance issues. It quantifies the overlap between predicted and ground truth segmentation results, proving particularly effective for objects with ambiguous boundaries. Commonly used to measure accuracy in target region delineation, it is suitable for handling small or uneven target regions as follows in Equation 13:

$$DSC\left(P,G\right)=\frac{2\times |P\cap G|}{\left|P|+|G\right|}$$ (13)

HD (Hausdorff Distance) serves as a distance measure for assessing the similarity between two sets and is frequently employed to evaluate model performance in image segmentation tasks. It proves especially valuable in medical image segmentation, enabling the quantification of disparities between predicted and actual segmentations. By capturing the maximum difference between true and predicted segmentation results, the computation of Hausdorff Distance is well-suited for evaluating the performance of segmentation models in boundary regions, as shown in Equation 14.

$$HD\left(A,B\right)=\max\left(\underset{a\in A}{\sup}\underset{b\in B}{\inf}d\left(a,b\right),\underset{b\in B}{\sup}\underset{a\in A}{\inf}d\left(a,b\right)\right)$$ (14)

mIoU (Mean Intersection over Union) is a region-based metric widely used in image segmentation, particularly in medical imaging, to evaluate the overlap between predicted and ground truth regions. It quantifies segmentation accuracy by calculating the ratio of intersection to union area, providing a robust measure of target region coverage, especially where precision is crucial, as shown in Equation 15.

$$mIoU\left(A,B\right)=\frac{1}{N}\overset{N}{\underset{i=1}{\sum }}\frac{\left|A_{i}\cap B_{i}\right|}{\left|A_{i}\cup B_{i}\right|}$$ (15)

ASD (Average Symmetric Surface Distance) is a boundary-based metric widely used in image segmentation, particularly in medical imaging, to quantify spatial discrepancies between predicted and ground truth surfaces. It measures the average distance between corresponding boundary points, providing precise evaluation of boundary delineation accuracy where localization is critical, as shown in Equation 16.

$$ASD\left(A,B\right)=\frac{1}{2}\left(\frac{1}{\left|S_{A}\right|}\underset{a\in S_{A}}{\sum }\underset{b\in S_{B}}{\min}d\left(a,b\right)+\frac{1}{\left|S_{B}\right|}\underset{b\in S_{B}}{\sum }\underset{a\in S_{A}}{\min}d\left(a,b\right)\right)$$ (16)

### 4.3 Experimental setup

In the KD process, the teacher model was specially selected as TransUnet (Chen J. et al., 2021), which is the representative model in medical image segmentation. We do not use the currently popular Vmamba (Liu et al., 2024) or MedSAM (Zhang et al., 2024), which rely on parallel feature processing and only symbolically maintain U-shaped structures. We also selected the typical and highly representative TransUnet, which has the classic U-shape structure, retaining the traditional Encoder-Decoder structure while incorporating the transformer structure into the model. TransUnet is a combination of Transformer and U-net architectures, with a total of 105,322,146 parameters. Leveraging the flexibility and attention mechanisms of Transformers, the unique amalgamation in TransUnet enables precise localization and rich contextual information, making it highly effective for various segmentation tasks. In the qualitative experiments, we exclusively employed TransUnet as the teacher model to control for variability and ensure consistent experimental conditions.

The student model we chose is derived by taking the initial TransUnet and reducing both the channel sizes of convolution modules and the parameters within the structure without pre-training. Our proposed student model not only reduces to 1/4 the size of each convolution layer but also removes the all-important transformer module. These operations led to the birth of the student model, with a total of only 6,678,009 parameters.

During the training process, two Nvidia RTX 3090 GPUs were utilized as computing devices, integrated with the Pytorch framework. For Fives dataset, the input size is 512x512 and the patch size of 4. For the Rose dataset, the input size is 256x256 and the patch is 2. For the Drive dataset, the input size is 256x256 and the patch is 2. Models are trained with SGD optimizer with a learning rate of 0.01, momentum of 0.9 and weight decay of 1e-4. We used 300 epochs for training on the three dataset.

### 4.4 Experimental results

The comparison of the proposed KD module with previous state-of-the-art (SOTA) methods. The overall experimental results were partitioned into two parts: quantitative results with state-of-the-art (SOTA) methods and ablation comparisons in U-structure.

#### 4.4.1 Quantitative result

##### 4.4.1.1 Results on the fives dataset

**FIGURE 5:**
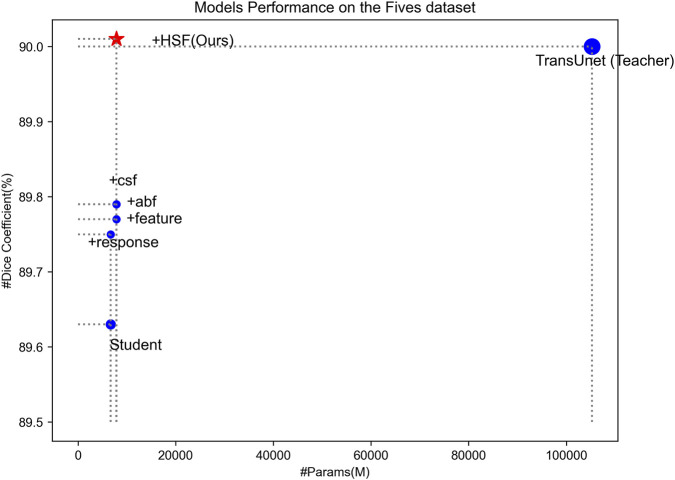
The number of parameters and accuracy of different KD methods on the Fives dataset. Blue dot denotes the student model, the student with other distillation methods and other high-performance models. Red pentagram denotes the model distilled by our proposed distillation method, HSF. In comparison with TransUnet serves as a teacher, we are surprised to find that the accuracy can outperform it with a significant reduction in the number of parameters. The proposed method can efficiently increase segmentation accuracy with few additional parameters in the training process.

To further validate the efficacy and computational efficiency of the proposed HSF module, we conducted extensive experiments on the FIVES retinal vessel segmentation dataset. As summarized in Table 1 and Figure 5, the HSF-integrated student model demonstrated superior performance across all primary segmentation metrics compared to other state-of-the-art (SOTA) distillation frameworks and recent lightweight architectures. Specifically, the HSF-distilled student model achieved a Dice Similarity Coefficient (DSC) of 90.01%, which not only surpassed the baseline Student (Unet) at 89.63% and competitive benchmarks such as MobileNetV2-UNet (87.75%) and the Vision Transformer-based SwinUnet (76.01%), but also slightly exceeded the performance of the Teacher (TransUnet) at 90.00%. While the recent MambaUNet achieved a marginally higher DSC (90.47%), our HSF-distilled model delivers highly comparable diagnostic fidelity while operating at a mere fraction of MambaUNet’s computational cost. Furthermore, our method exhibited significant improvements in boundary precision, yielding a Hausdorff Distance (HD) of 5.95 and a mean Intersection over Union (mIoU) of 82.84%, effectively outperforming established modules such as CSF (HD: 6.57) and the conventional Response-based KD (HD: 8.04).

**TABLE 1:** Comprehensive efficiency and performance analysis on FIVES Dataset. We compare FLOPs, memory footprint, inference latency, and segmentation metrics. HSF achieves the best efficiency-accuracy trade-off, with 18.4% lower FLOPs and 16.3% faster inference than CSF while maintaining superior DSC performance.

Model (+KD module)	KD	FLOPs(G)	Params(M)	Memory (MB)	Latency (ms)	DSC ↑	HD ↓	mIoU ↑	ASD ↓
Teacher (TransUnet)	N/D	593.47	105.30	420.5	45.2	90.00	5.63	82.71	83.23
ResUnet (Diakogiannis et al., 2020)	N/D	1485.92	13.04	58.2	28.6	84.07	17.40	74.74	123.73
MobileNetV2-UNet (Sandler et al., 2018)	N/D	8.26	3.98	15.33	14.63	87.75	6.62	79.12	96.02
MambaUNet (Wang et al., 2024)	N/D	209.81	30.27	115.54	34.50	90.47	5.83	83.58	76.10
SwinUnet (Cao et al., 2022)	N/D	-	27.17	110.09	19.38	76.01	11.93	62.56	2.6203
Student (Unet)	N/D	75.48	6.68	32.5	8.4	89.63	7.40	82.28	128.55
+KD (Hinton et al., 2015)	Response	75.48	6.68	35.8	8.6	89.75	8.04	82.49	82.16
+Feature (Chen P. et al., 2021)	Feature	134.52	7.85	68.4	15.2	89.79	6.28	82.50	126.06
+ABF (Chen P. et al., 2021)	Related	134.58	7.85	68.5	15.4	89.77	6.68	82.50	81.27
+CSF (Liu et al., 2023a)	Related	134.53	7.89	68.6	15.3	89.92	6.57	82.74	73.82
+HSF(Ours)	Related	109.80	8.14	52.3	12.8	90.01	5.95	82.84	75.55

Beyond segmentation accuracy, the HSF framework achieved a remarkable reduction in computational complexity, explicitly addressing the hardware constraints of real-world clinical deployment. The student model equipped with the HSF module utilizes only 8.14 M parameters, representing approximately 7.73% of the Teacher model’s total parameter count (105.3 M). More importantly, it drastically minimizes operational overhead across all critical metrics: FLOPs are slashed from 593.47 G to 109.80 G, memory footprint drops from 420.5 MB to 52.3 MB, and inference latency is accelerated from 45.2 ms to 12.8 ms relative to the Teacher. Crucially, when compared to MambaUNet, our HSF framework reduces the parameter count by over 73% (8.14 M vs. 30.27 M), cuts FLOPs by nearly half (109.80 G vs. 209.81 G), and achieves a 2.6 × speedup in inference latency (12.8 ms vs. 34.50 ms). This definitively proves that HSF’s slight trade-off in DSC yields immense efficiency gains. Even against the highly competitive CSF distillation method, HSF delivers an 18.4% reduction in FLOPs and 16.3% faster inference while maintaining superior precision, successfully establishing an optimal efficiency-accuracy trade-off for resource-constrained environments.

**FIGURE 6:**
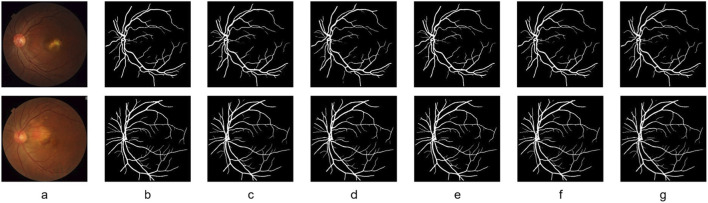
Semantic segmentation Dices of our HSF compared others KD methods on Fives datasets. The columns from left to right represent: **(a)** Original Image **(b)** Ground Truth **(c)** Student **(d)** Feature **(e)** ABF **(f)** CSF and **(g)** HSF (Ours).

Qualitative analysis further supports these quantitative findings. As illustrated in Figure 6, the FIVES dataset presents a significant challenge due to its intricate and attenuated vascular structures. While conventional heavy models, Vision Transformers (e.g., SwinUnet), or excessively compressed lightweight networks often overlook localized morphological details in low-contrast regions, the HSF module leverages specialized gating and fusion mechanisms to guide the lightweight student in capturing fine-grained vascular features. Visual inspection of the segmented outputs (a-g) reveals that the HSF module facilitates the generation of more continuous and topologically accurate vessel skeletons, closely aligning with the Ground Truth (b). The balanced performance in HD (5.95) and Average Surface Distance (ASD: 75.55) confirms the module’s capability in preserving structural connectivity and reducing noise, thereby providing high-fidelity representations of complex biological vasculatures.

##### 4.4.1.2 Results on the rose dataset

To evaluate the generalizability of our framework across diverse vascular morphologies, we further assessed the HSF module on the ROSE dataset. The experimental results, detailed in Table 2, demonstrate that our distillation method consistently enhances the performance of the lightweight student model while maintaining exceptional parameter efficiency. The HSF-distilled student model achieved a Dice Similarity Coefficient (DSC) of 77.92% and a mean Intersection over Union (mIoU) of 64.12%, significantly narrowing the performance gap with the Teacher model (TransUnet), which achieved a DSC of 78.30% and mIoU of 64.63%. Notably, the student model attained these results using only 7.73% of the Teacher’s parameters, representing a substantial reduction in computational requirements compared to the original architecture.

**TABLE 2:** Comparative of parameters efficiency with related model on the Rose dataset.

Model (+KD module)	KD	Avg params (%)	DSC ↑	mIoU ↑
Teacher (TransUnet)	N/D	100.00%	78.30	64.63
Student (Unet)	N/D	6.34%	76.46	62.10
+KD (Hinton et al., 2015)	+Response	6.34%	77.11	63.00
+Feature (Chen P. et al., 2021)	+Feature	7.45%	77.54	63.61
+ABF (Chen P. et al., 2021)	+Related	7.46%	77.59	63.67
+CSF (Liu et al., 2023a)	+Related	7.50%	77.71	63.83
+HSF(Ours)	+Related	7.73%	77.92	64.12

In comparison with other state-of-the-art distillation modules, the HSF method demonstrated superior performance. It outperformed the conventional Response-based KD (+0.81% DSC), the Feature-based module (+0.38% DSC), and relation-based competitors such as ABF (+0.33% DSC) and CSF (+0.21% DSC). This consistent improvement suggests that the specialized gating and fusion mechanisms within our HSF module are particularly effective at reconstructing complex relational knowledge necessary for characterizing intricate vascular networks.

**FIGURE 7:**
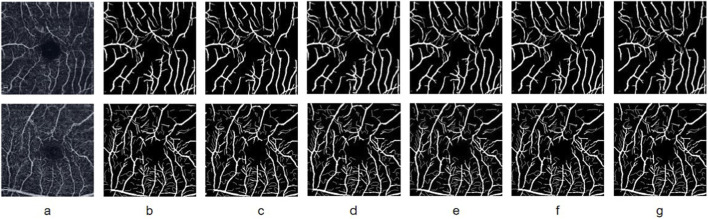
Semantic segmentation Dices of our HSF compared others KD methods on Rose datasets. The columns from left to right represent: **(a)** Original Image **(b)** Ground Truth **(c)** Student **(d)** Feature **(e)** ABF **(f)** CSF and **(g)** HSF (Ours).

The qualitative impact of our distillation framework is further illustrated in Figure 7, which provides a visual comparison of segmentation outputs. Retinal images in the ROSE dataset often contain low-contrast, fine-branching vessels that pose significant challenges for automated segmentation. As shown in the figure, while the baseline student model often suffers from vessel discontinuity and missed distal branches, the student model distilled with the HSF module produces a significantly more coherent vascular skeleton. By effectively guiding the student to focus on essential morphological features, our method ensures high-fidelity connectivity and structural integrity, closely mirroring the ground truth observations. These results collectively indicate that the HSF module provides a robust and efficient solution for high-precision biological image synthesis and analysis.

##### 4.4.1.3 Results on the DRIVE dataset

###### 4.4.1.3.1 Validation on Established Clinical Benchmarks

To rigorously address the dataset limitations and further validate the universality of our proposed framework, we conducted additional comprehensive evaluations on the DRIVE dataset, the most established benchmark in retinal vessel segmentation. As demonstrated in Table 3, the HSF-equipped student model consistently outperforms existing knowledge distillation paradigms. Specifically, our method achieves a DSC of 70.96% and an mIoU of 55.05%. This yields a substantial +1.62% DSC improvement over the baseline Student model (69.34%), effectively narrowing the performance gap with the computationally expensive Teacher network (71.81%). When compared against other state-of-the-art distillation approaches, HSF demonstrates superior morphological preservation, surpassing both conventional response-based KD (DSC: 69.65%) and the competitive CSF module (DSC: 70.82%). Crucially, this robust performance on a completely independent and distinct device-captured dataset (DRIVE) is achieved while utilizing a mere 7.73% of the Teacher’s total parameters. These results strongly confirm that our morphology-aware distillation strategy is not overfitted to the specific imaging characteristics of the FIVES or ROSE datasets, but rather serves as a universally applicable architecture for diverse ophthalmic imaging scenarios.

**TABLE 3:** Comparative of parameters efficiency with related model on the DRIVE dataset.

Model (+KD module)	KD	Avg params (%)	DSC ↑	mIoU ↑
Teacher (TransUnet)	N/D	100.00%	71.81	56.08
Student (Unet)	N/D	6.34%	69.34	53.16
+KD (Hinton et al., 2015)	+Response	6.34%	69.65	53.51
+Feature (Chen P. et al., 2021)	+Feature	7.45%	70.57	54.61
+ABF (Chen P. et al., 2021)	+Related	7.46%	70.95	55.05
+CSF (Liu et al., 2023a)	+Related	7.50%	70.82	54.90
+HSF(Ours)	+Related	7.73%	70.96	55.05

#### 4.4.2 Ablation comparisons

##### 4.4.2.1 Effect of KD Modules in U-shaped Model

In Table 4, we provide a comprehensive comparison between different U-shaped knowledge distillation (KD) frameworks namely EncodeKD, DecodeKD, and NormalKD (the integration of both Encode and Decode KD) evaluating their performance on the FIVES dataset using DSC, mIoU, and model parameter counts. Our empirical findings indicate that while NormalKD generally yields higher performance across various KD modules, DecodeKD consistently demonstrates superior efficacy compared to EncodeKD in retinal vessel segmentation.

**TABLE 4:** Comparison with other distillations methods in different U-shaped KD framework on the fives dataset.

KD module	Encode	Decode	Params (M)	DSC	mIoU
Student	-	-	6.68	89.63	82.28
+ Feature (Chen P. et al., 2021)	✓	×	7.69	89.73	82.44
	✓	✓	8.86	89.83	82.55
	×	✓	7.84	89.79	82.50
+ ABF (Chen P. et al., 2021)	✓	×	7.69	89.88	82.63
	✓	✓	8.87	89.86	82.60
	×	✓	7.85	89.77	82.50
+ CSF (Liu et al., 2023a)	✓	×	7.72	89.87	82.62
	✓	✓	8.96	89.79	82.52
	×	✓	7.89	89.92	82.74
+ HSF (Ours)	✓	×	7.89	89.88	82.67
	✓	✓	9.46	89.91	82.73
	×	✓	8.14	90.01	82.84

However, a critical analysis of the trade-off between performance gains and computational costs reveals that the incremental improvements offered by NormalKD often do not justify the additional parameter overhead. Notably, when utilizing our proposed HSF method within the DecodeKD framework, we achieved a peak DSC of 90.01% and a mIoU of 82.84%. This configuration not only represents the best overall segmentation performance among all tested KD methods but also maintains a highly efficient parameter profile (8.14 M) compared to more complex NormalKD implementations. For instance, our HSF-based DecodeKD outperformed its NormalKD counterpart (DSC: 89.91%, Params: 9.46 M) while utilizing significantly fewer parameters. This suggests that the HSF module is particularly adept at refining high-level semantic features during the decoding stage, enabling the student model to capture intricate vascular structures with higher fidelity and lower redundancy than conventional dual-stage distillation approaches.

##### 4.4.2.2 Effect of HSF Submodules for the Effectiveness of MRD Framework

To dissect the specific contribution of each architectural component within our proposed HSF module, we conducted a series of ablation studies on the FIVES dataset, as summarized in Table 5. Our analysis proceeds by incrementally adding key components: Fuse Cross Stage Feature (FCSF), Selective Fusion (SF), and Residual Gating (RG), to a baseline feature fusion framework. Starting with the baseline Unet model (DSC: 89.63%, mIoU: 82.28%), a simple cross-stage feature distillation (marked as ×,×,× ) yields a Dice score of 89.79% and an mIoU of 82.50%.The introduction of the FCSF module (✓,×,×) further enhances the performance to 89.92% DSC and 82.75% mIoU, underscoring the benefit of creating a more sophisticated, shared contextual representation prior to fusion. Interestingly, the subsequent addition of the SF mechanism (✓,✓,×) , designed to selectively amplify critical features, results in a performance of 89.96% DSC and 82.77% mIoU. Consistent with observations in complex synthesis tasks, this suggests that an unrestrained selective fusion mechanism may become overly aggressive, potentially forcing the student to suppress its own learned features in favor of the teacher’s specific patterns.The final integration of the Residual Gating (RG) mechanism (✓,✓,✓) resolves this imbalance and achieves the optimal performance of 90.01% DSC and 82.82% mIoU. Given the strict morphological fidelity required in ophthalmic imaging, the RG acts as a crucial regulator. It allows the student model to absorb the teacher’s structural wisdom while preserving the integrity of its own multi-scale feature representations. This synergistic interplay ensures high-quality segmentation of the intricate retinal vasculature, preventing the topological information loss occasionally observed when relying on SF alone.

**TABLE 5:** Ablation study on HSF submodules for the effectiveness of HSF on the fives dataset.

FCSF	SF	RG	DSC ( ↑ )	mIoU ( ↑ )
Baseline			89.63	82.28
×	×	×	89.79	82.50
✓	×	×	89.92	82.75
✓	✓	×	89.96	82.77
✓	✓	✓	90.01	82.82

FCSF: Fuse Cross Stage Feature module. SF: Selective Fusion module. RG: Residual Gating module.

##### 4.4.2.3 Effect of KD Modules with Feature Map Distillation Loss

A pivotal aspect of our framework is the synergy between the HSF module and the Hierarchical Context Loss (HCL). We validated this by comparing HCL with the standard Mean Squared Error (MSE) loss on the FIVES dataset, as presented in Table 6. The experimental results consistently indicate that the combination of HSF and HCL yields the superior performance compared to traditional pixel-wise objectives.

**TABLE 6:** Ablation experiments for KD modules with feature map distillation loss on the fives dataset.

Setting	DSC( ↑ )	mIoU ( ↑ )
Baseline	89.63	82.28
Baseline + CSF + MSE	89.91	82.67
Baseline + CSF + HCL	89.92	82.74
Baseline + HSF + MSE	89.94	82.73
Baseline + HSF + HCL	90.01	82.82

Specifically, when utilizing the HSF module with MSE loss, the model achieves a Dice score of 89.94% and an mIoU of 82.73%. While MSE can effectively capture local feature similarities, it often misses the broader structural and relational information essential for continuous vessel segmentation. In contrast, switching to the HCL objective provides a substantial performance boost, with the HSF + HCL configuration reaching the peak results of 90.01% DSC and 82.82% mIoU. This configuration significantly outperforms the CSF + HCL setup, which yields 89.92% DSC.

The HSF module is explicitly designed to generate feature maps that are structurally and hierarchically rich. HCL, through its contrastive nature, excels at evaluating the global “big picture” of the synthesized features. Consequently, HSF and HCL are highly complementary: HSF generates the structurally meaningful features required for vascular analysis, while HCL provides the ideal guidance to ensure these features are arranged in the correct global configuration. This synergy is paramount for maintaining the connectivity of the retinal microvasculature, providing a high-fidelity segmentation solution suitable for cell-level developmental studies.

##### 4.4.2.4 Clinical Relevance and Failure Case Analysis

**FIGURE 8:**
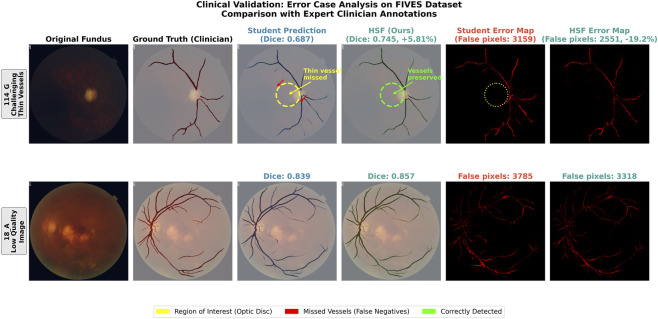
Clinical Validation on FIVES Dataset with Expert Annotations. Comparative segmentation results on two representative clinical cases from the FIVES dataset. (200 test cases with gold-standard clinician annotations).

To further assess the clinical applicability and robustness of the proposed method, we conducted a visual error analysis on representative challenging cases from the FIVES dataset, comparing the predictions against expert clinician annotations (Figure 8). Specifically, in scenarios characterized by extremely thin vessels near the optic disc (e.g., Case 114_G), the baseline Student model exhibits severe topological fractures, failing to detect fine vascular branches (Dice: 0.687). Conversely, the HSF method effectively preserves these delicate structures, yielding a substantial +5.81% improvement in the Dice score. Furthermore, when evaluated on low-quality clinical images with suboptimal contrast and sharpness (e.g., Case 18_A), HSF demonstrates enhanced robustness (Dice: 0.857 vs. 0.839). As illustrated by the error maps (where false negatives are highlighted in red), the hierarchical multi-scale feature fusion in HSF significantly reduces vessel disconnections and missed microvasculature, affirming its potential for reliable deployment in resource-constrained clinical environments.

## 5 Discussion

This study presents a specialized strategy for deployment-oriented ophthalmic image analysis, specifically targeting the challenge of maintaining vascular fidelity in lightweight segmentation models. While deep learning has significantly advanced the automated analysis of retinal vessels, a critical gap remains between high-performance “heavy” models and the resource-constrained requirements of clinical edge devices. Our work addresses this by moving beyond generic model compression, introducing a task-specific solution that prioritizes vascular morphology and topological continuity across Fundus photography and OCT Angiography (OCTA).

The proposed Hierarchical Structure Fusion (HSF) module, serving as the core engine within our Morphology-Aware Reconstruction Distillation (MRD) framework, demonstrates substantial improvements across ophthalmic imaging modalities. Our approach excels by resolving the inherent tension between model compression and structural fidelity through two key mechanisms:

1. Morphology- Feature Fusion: HSF effectively combines hierarchical features from different stages of the student model while strictly preserving the spatial characteristics necessary for delineating thin, continuous vessels. This fusion strategy addresses a common limitation in existing methods, which often dilute pre-fusion spatial details, leading to fractured segmentations.
2. Targeted Topological Distillation: By utilizing a residual gating mechanism, our method enables precise, selective feature distillation. Instead of forcing the student to memorize redundant pixel statistics, this targeted approach guides the model to synthesize the structural “grammar” of the vasculature, leading to highly efficient knowledge reconstruction.

The superiority of our framework over existing KD modules, as evidenced by our empirical results (Tables 1, 2), is driven by several theoretical and biological advantages:

- Protection of High-Frequency Biological Details: Unlike traditional KD methods that often lose delicate information during the reconstruction process, HSF’s residual gating mechanism enables adaptive feature recalibration. This ensures the preservation of critical low-level spatial features (e.g., micro-capillaries) from the original architecture. This preservation is vital in clinical ophthalmology, where the continuity of fine-grained vessels directly impacts the diagnosis of neurodegenerative and systemic diseases.
- Robustness to Modality-Specific Artifacts: The selective fusion component of HSF allows for a nuanced integration of features. By dynamically adjusting the importance of different feature levels, HSF successfully isolates true vascular signals from profound modality-specific noise, such as illumination variance in Fundus photography and projection artifacts in OCTA.
- Decoder-Centric Computational Efficiency: Our framework’s explicit focus on distilling key features from the decoder perfectly aligns with the U-shaped network’s role as a topological reconstruction engine. Full-stage distillation often leads to attention dispersion; by contrast, targeting the decoder reduces computational overhead while strictly teaching the student how to synthesize coherent semantic and spatial information.

Despite these advancements, it is important to acknowledge the limitations of our current approach, which highlight promising directions for future research. First, our MRD framework and the HSF module are fundamentally tailored for the specific encoder-decoder symmetry of U-shaped convolutional neural networks; extending these morphology-aware principles to fundamentally different paradigms, such as pure Vision Transformers (ViTs) or diffusion-based generative models, will require significant structural adaptations. Furthermore, while our model generalizes well across the diverse noise profiles of the Fives and Rose datasets, the universal challenge of medical data scarcity and cross-center variability remains. Integrating reconstruction learning or unsupervised target domain adaptation strategies into our lightweight models is urgently needed to ensure robust, large-scale deployment across diverse hospital networks and varying scanner protocols. Additionally, the current implementation faces computational bottlenecks when scaled to 3D volumetric segmentation tasks. Even when distillation is strictly localized to decoder features via MRD, the frequent computation and hierarchical fusion of 3D feature maps remain resource-intensive, underscoring the need for developing ultra-efficient, sparse 3D distillation techniques optimized for volumetric ophthalmic data. Finally, although the HSF module demonstrates robust effectiveness through its residual gating mechanism, its relative performance gains can plateau. Similar to other state-of-the-art distillation methods, when applied to relatively simple segmentation tasks where the inherent performance gap between the teacher and student is already marginal, the benefits of distillation are limited. This emphasizes the necessity of dynamically calibrating knowledge distillation strategies based on the intrinsic anatomical complexity of the task. Future efforts will focus on extending the MRD framework to broader architectures and 3D modalities, exploring pathways to further reduce computational demands while rigorously maintaining biological and topological accuracy.

## 6 Conclusion and future work

In this paper, we presented Morphology-Aware Reconstruction Distillation (MRD), a novel framework explicitly designed to address the deployment bottleneck of deep learning in computational ophthalmology. Accurate segmentation of the retinal microvasculature is a critical prerequisite for diagnosing ocular and systemic diseases. However, unlike conventional knowledge distillation (KD) methods that treat U-shaped architectures as generic feature extractors (Chen P. et al., 2021; Liu et al., 2023a; Qi et al., 2022; Cho and Hariharan, 2019), MRD specifically targets the decoder to preserve the fundamental biological priors of the retinal vascular topology. Central to this framework is the Hierarchical Structure Fusion (HSF) module, which adaptively integrates multi-scale features to explicitly filter out profound ophthalmic imaging noise—such as severe illumination variance in Color Fundus (CF) photography and inherent projection artifacts in Optical Coherence Tomography Angiography (OCTA).

Our extensive experimental results provide compelling evidence of the framework’s effectiveness across these diverse ophthalmic modalities. Notably, on the Fives dataset (Fundus Photography), our highly compact student model achieved a remarkable Dice score of 90.01% after distillation, successfully outperforming its heavily parameterized teacher, TransUNet (90.00%), as well as other large networks like ResUNet (84.07%) (Table 1). This performance leap validates our approach and suggests a task-specific distillation strategy in retinal image analysis: lightweight, structurally-aware distilled models can achieve, and even surpass, the morphological fidelity of their complex counterparts, effectively preventing the fragmentation of fine capillaries.

By proving that clinical accuracy does not strictly necessitate massive computational overhead, our framework opens up new possibilities for deploying advanced automated segmentation algorithms directly onto portable fundus cameras and point-of-care OCTA devices. While our current study focuses on 2D convolutional neural networks, adapting these structure-aware principles to emerging architectures, such as Vision Transformers (Liu et al., 2023a) and 3D volumetric OCTA models, presents a clear direction for future research. Continued development in this direction promises to democratize access to state-of-the-art retinal imaging analysis, ultimately accelerating precise screening workflows and improving patient outcomes in global eye care.

## Data Availability

Publicly available datasets were analyzed in this study. This data can be found here: ROSE Dataset (OCT Angiography): https://imed.nimte.ac.cn/dataofrose.html, FIVES Dataset (Color Fundus Photography): https://figshare.com/articles/figure/FIVES_A_Fundus_Image_Dataset_for_AI-based_Vessel_Segmentation/19688169/1.
